# Longitudinal microbiome investigation throughout prion disease course reveals pre- and symptomatic compositional perturbations linked to short-chain fatty acid metabolism and cognitive impairment in mice

**DOI:** 10.3389/fmicb.2024.1412765

**Published:** 2024-06-11

**Authors:** Marco Losa, Yasser Morsy, Marc Emmenegger, Salomon M. Manz, Petra Schwarz, Adriano Aguzzi, Michael Scharl

**Affiliations:** ^1^Department of Gastroenterology and Hepatology, University Hospital Zurich, University of Zurich, Zürich, Switzerland; ^2^Institute of Neuropathology, University Hospital Zurich, University of Zurich, Zürich, Switzerland; ^3^Institute of Clinical Immunology, University Hospital Basel, Basel, Switzerland

**Keywords:** gut–brain–microbiome axis, microbiome, fecal 16S rRNA seq, metabolism, short-chain fatty acids, prion disease, neuroinflammation, intracerebral pathologies

## Abstract

Commensal intestinal bacteria shape our microbiome and have decisive roles in preserving host metabolic and immune homeostasis. They conspicuously impact disease development and progression, including amyloid-beta (Aβ) and alpha (α)-synuclein pathology in neurodegenerative diseases, conveying the importance of the brain–gut–microbiome axis in such conditions. However, little is known about the longitudinal microbiome landscape and its potential clinical implications in other protein misfolding disorders, such as prion disease. We investigated the microbiome architecture throughout prion disease course in mice. Fecal specimens were assessed by 16S ribosomal RNA sequencing. We report a temporal microbiome signature in prion disease and uncovered alterations in Lachnospiraceae, Ruminococcaceae, Desulfovibrionaceae, and Muribaculaceae family members in this disease. Moreover, we determined the enrichment of Bilophila, a microorganism connected to cognitive impairment, long before the clinical manifestation of disease symptoms. Based on temporal microbial abundances, several associated metabolic pathways and resulting metabolites, including short-chain fatty acids, were linked to the disease. We propose that neuroinflammatory processes relate to perturbations of the intestinal microbiome and metabolic state by an interorgan brain–gut crosstalk. Furthermore, we describe biomarkers possibly suitable for early disease diagnostics and anti-prion therapy monitoring. While our study is confined to prion disease, our discoveries might be of equivalent relevance in other proteinopathies and central nervous system pathologies.

## Introduction

Microorganisms, especially host-resident bacteria that colonize mammalian intestines in large quantities, are collectively termed as gut microbiome. They are paramount players of the gut–brain–microbiome axis (Pickard et al., 2017). Their identified functions have vastly increased in the last decade and range from the metabolism of essential nutrients to regulation of cellular development and prevention of host dysbiosis and pathogen invasion (Bradford et al., 2017). Many reports describe that physiological processes of the central nervous system (CNS) rely on gut microbiota. There is a complex bidirectional and interorgan crosstalk between the brain and the intestine by a variety of different systems including endocrine, immune, autonomic, enteric nervous systems and microbiota-derived micro- and macromolecules (Lach et al., 2018; Lai et al., 2021). Microbiota exert their effects through short-chain fatty acids (SCFAs), branched-chain amino acids, peptidoglycans and peptides, indoxyl sulfate, trimethylamine-*N*-oxide, tryptophan metabolism, blood–brain barrier permeability modification, neurotransmitters modulation, accurate microglia function control, and vagus nerve excitation (Braniste et al., 2014; Erny et al., 2015; Strandwitz, 2018; Cryan et al., 2019; Colombo et al., 2021; Lai et al., 2021). Microbiota-produced SCFAs are believed to influence microglia activity via meningeal immune cells (Rothhammer et al., 2016; Martin-Pena and Tansey, 2023; Seo et al., 2023). In addition to peripheral immune homeostasis, the presence of a complex microbiome and SCFA receptors is crucial for proper function and maturation of microglia (Erny et al., 2015) and regulation of neuronal circuits (Yu and Hsiao, 2021). There is increasing evidence that the microbiome and dysbiosis largely influence several conditions such as autism, anxiety, schizophrenia, brain tumor pathogenesis (i.e., tumor growth), multiple sclerosis (MS), as well as protein misfolding disorders such as amyotrophic lateral sclerosis (ALS), Parkinson’s disease (PD), Alzheimer’s disease (AD), and prion disease (PrD) via the gut–brain–microbiome axis (Minter et al., 2016; D'Argenio and Sarnataro, 2019; Mehrian-Shai et al., 2019; Montalban-Arques et al., 2021; Montella et al., 2021; Claudino Dos Santos et al., 2023; Heston et al., 2023; Mahbub et al., 2024). Furthermore, targeting of microbiota-derived SCFAs has been proven to effectively modulate and potentiate the immunotherapeutic response in colorectal cancer therapy *in vivo* (Montalban-Arques et al., 2021). Although there is a consensus that gut microbiome composition can be altered in patients with AD, PD, or ALS (Martin-Pena and Tansey, 2023), microbiome perturbations and the neuro-immunological crosstalk along the gut–brain–microbiome axis in prion disease are underreported and still poorly understood.

Prion diseases (PrDs) are incurable protein misfolding disorders characterized by a long incubation period (years to decades) and a rapid disease progression upon symptom onset (Brown et al., 1986). These diseases are caused by a single cellular membrane protein, the prion protein (PrP^C^), that undergoes a pathological transformation into its aggregation-prone form (PrP^Sc^). PrP^Sc^ self-assembles and propagates into neurotoxic extracellular PrP aggregates accommodated by microglia and astrocyte activation and neuroinflammation (Aguzzi and Weissmann, 1998; Scheckel and Aguzzi, 2018; Scheckel et al., 2020). Toxic PrP aggregates seed into most brain regions, including the brain stem, affecting neuronal circuits as well as efferent and afferent neuronal signaling thereof (Mirabile et al., 2015; Carta and Aguzzi, 2022). However, it is unclear whether brain (stem) pathology could relate to microbiome architectural changes, and if so, whether this even happens before the onset of disease symptoms. Recent promising human and rodent data report prion-specific preclinical hippocampal transcriptional (Sorce et al., 2020) and peripheral muscle tissue metabolism (Caredio et al., 2023) changes. This indicates that the discovery and clinical employment of early PrD biomarkers are feasible. Moreover, it may be that such biomarkers are also applicable in other neurodegenerative diseases sharing similar pathomechanisms (Barnham et al., 2006).

The question we therefore addressed was whether prion disease and neuroinflammation are associated with alterations in the microbiome composition in a temporal manner. We therefore investigated the gut microbiome of prion-infected animals throughout the entire prion disease course by fecal 16S ribosomal RNA sequencing.

Here, we report a temporal microbiome signature of prion disease. We detected pre- as well as post-symptomatic microbiome perturbations that might lead to the development of novel, readily accessible biomarkers and alternative therapeutic approaches. Our data provide novel insights into the brain–gut–microbiome axis and its dynamics in prion disease. Our study might therefore have broader clinical implications in a variety of proteinopathies and intracerebral pathologies.

## Results

### Temporal shift in microbiome composition along the prion disease course

**Figure 1:**
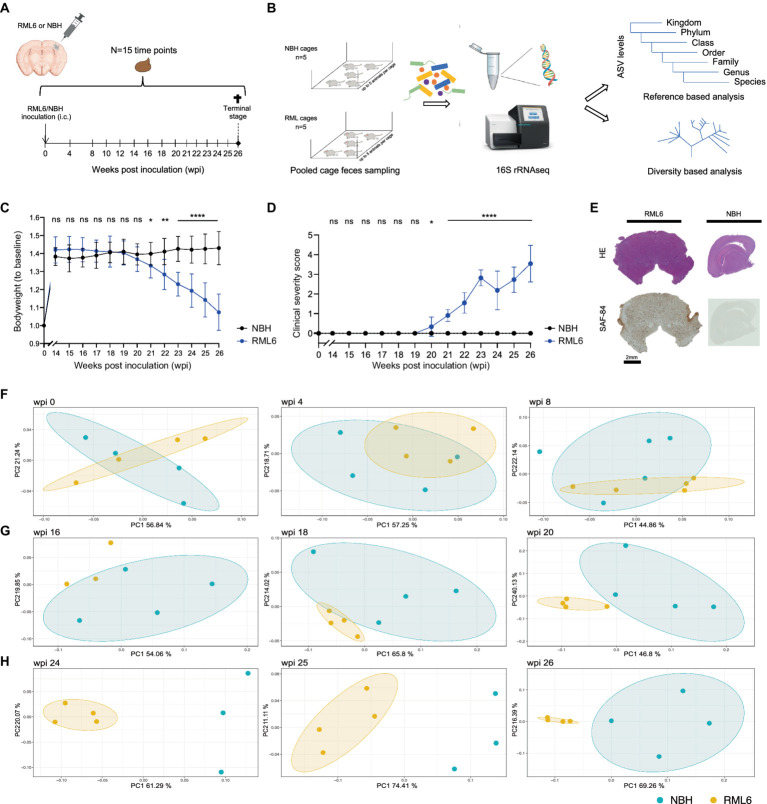
Identification of disease-specific microbiome alterations in prion disease. **(A)** Intracerebral inoculation and feces sampling scheme throughout the course of prion disease. **(B)** Overview of the experimental pipeline shows 16S rRNA sequencing and consecutive reference as well as diversity-based analysis. **(C)** Body weight development among the experimental animals. Of note: Prion-infected animals (RML6 inoculated) show bodyweight loss from 19 wpi. Two-tailed Student’s *t*-test was performed for each time point. **(D)** Clinical severity score over time shows the clinical course of experimental animals. Of note: Clinical symptoms of experimental animals are congruent with the body weight loss displayed in **(C)**. Two-tailed Student’s *t*-test was performed. **(E)** Representative HE-stained and SAF84 IHC brain sections (coronal for RML6 and sagittal for NBH) of experimental animals at the terminal stage show diffuse prion aggregation throughout the brain, including the brainstem (left panel). SAF84 positivity appears brown in IHC. No SAF84 positivity in NBH control mice. **(F–H)** Principal coordinate analysis on sequencing data showing weighted UniFrac beta distance matrix from three different time points **(F)** at the early stage (wpi 0, 4, 8), **(G)** at the middle symptomatic stage (wpi 16, 18, 20), and **(H)** at the end stage (wpi 24, 25, 26) of prion disease course. A difference between the experimental groups can be detected from wpi 16. A single data point represents pooled fecal sequencing data originating from one animal cage. By default, four cages per group were used. Of note, sequencing data sets from time points wpi16 (RML6), wpi24 (NBH), and wpi25 (NBH) did not pass quality control, resulting in the absence of respective vector illustrations. *p < 0.05; **p < 0.01; ****p < 0.0001; ns: not significant.

Microbiota have been shown to influence Aβ aggregation and α-synuclein pathology in AD and PD via microglia modulations, respectively (Erny et al., 2015; D'Argenio and Sarnataro, 2019; Colombo et al., 2021; Claudino Dos Santos et al., 2023). As microglia activation (Aguzzi and Zhu, 2017) and brain stem pathology (Mirabile et al., 2015; Reis et al., 2015) are hallmarks in prion disease and correspond to the clinical phenotype, we hypothesized that the microbiome signature changes during the prion disease course. To this end, we first investigated whether prion-infected animals differ from control mice regarding microbiome composition throughout the course of prion disease. To do so, we employed a well-studied murine model of prion disease and intracerebrally injected Rocky Mountain Laboratory 6 (RML6) prion strain and non-infectious brain homogenate (NBH) into wild-type male mice of similar age (Supplementary Table S1; Sorce et al., 2020; Frontzek et al., 2022). Each experimental group consisted of *n* = 21 animals (total *n* = 42 mice) that were initially co-housed in five cages per experimental group (Supplementary Table S2). Pooled fecal specimen derived from each cage containing up to five mice were collected with a weekly interval from baseline (prior to any injections) to terminal stage, denominated as weeks post-inoculation (wpi; see Figure 1A). Consecutively, we processed the fecal specimen from RML6 and NBH-treated mice from *n* = 15 time points and performed 16S V4 ribosomal RNA sequencing and phylogenetic reconstruction (Figure 1B) to characterize the microbiome landscape and alterations between the two conditions over time. After data quality check and denoising, amplicon sequence variants (ASVs) were generated upon paired read merging (Callahan et al., 2016). The sequencing data had sufficient depth to capture the most expected features and was consecutively analyzed using a diversity- as well as reference-based approach. Utilizing this murine model of prion disease leads to first clinical symptoms at approximately 20 weeks’ post-inoculation (Sorce et al., 2020; Caredio et al., 2023). This was assessed by body weight loss and a clinical severity score (Figures 1C,D). To assess successful prion inoculation and propagation, we performed HE and SAF-84 immunohistochemistry on brain slices upon euthanasia and detected prion aggregates throughout the brain (Zhu et al., 2019; Frontzek et al., 2022; Figure 1E). We then went on and calculated both the microbiome diversity within (alpha diversity) and across (beta diversity) experimental groups. The former was calculated using the faith phylogenetic matrix to compute the richness and incorporate phylogenetic relations. The latter was calculated using the weighted UniFrac distance matrix to quantify the dissimilarity between communities (Diallo et al., 2007). We identified trends in the alpha diversity among microbiomes of experimental groups starting just before the onset of clinical symptoms (Supplementary Figure S1). By performing a principal coordinate analysis (PCoA) on all samples, divided into early, middle symptomatic, and end stages, we identified a progressive separation of the two experimental groups over time (Figures 1F–H and Supplementary Table S3). While in the early stage of disease no separation was observable (left to right, *p* = 0.830, 0.306, 0.298) (Figure 1F), the RML6 and NBH-treated groups gradually formed distinct clusters, starting from wpi 16–20 (left to right, *p* = 0.215, 0.033, 0.022) (Figure 1G), which was most pronounced toward the end stage of disease (left to right, *p* = 0.029, 0.029, 0.031) (Figure 1H). From these data, we conclude that the overall microbiome composition shifts in a temporal manner in mice inoculated with prions, starting around 12 weeks before symptom onset.

#### Compositional microbiome assessment identified enrichment of Lachnospiraceae and Ruminococcaceae in prion-diseased animals

**Figure 2:**
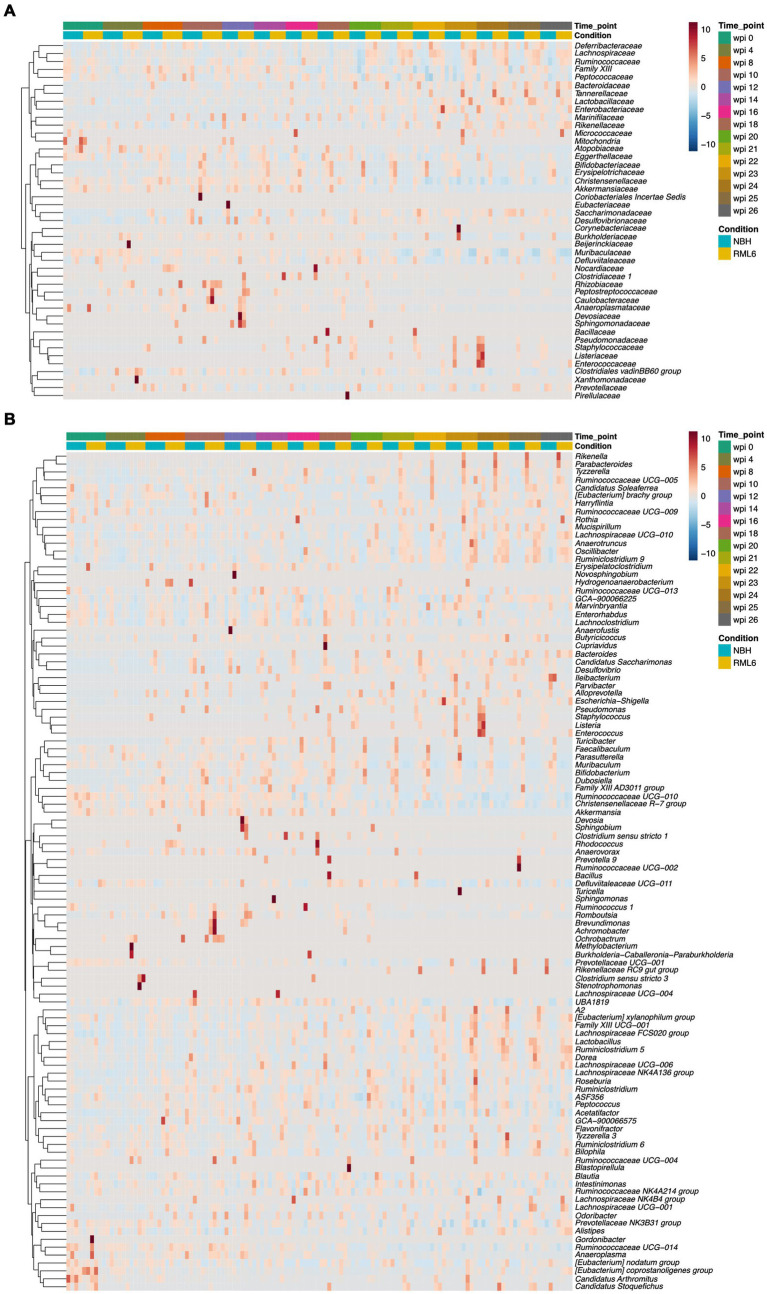
Detected families in experimental groups across selected disease time points. **(A)** The heat map displays the detected microbiome families and their abundance among experimental groups from baseline to terminal stage of the disease. Every time point contains the analysis of 10 fecal specimen. **(B)** The heat map displays the detected microbiome genera and their abundance among experimental groups across selected time points from baseline to terminal stage of the disease. Every time point contains the analysis of 10 pooled fecal cage specimen.

**Figure 3:**
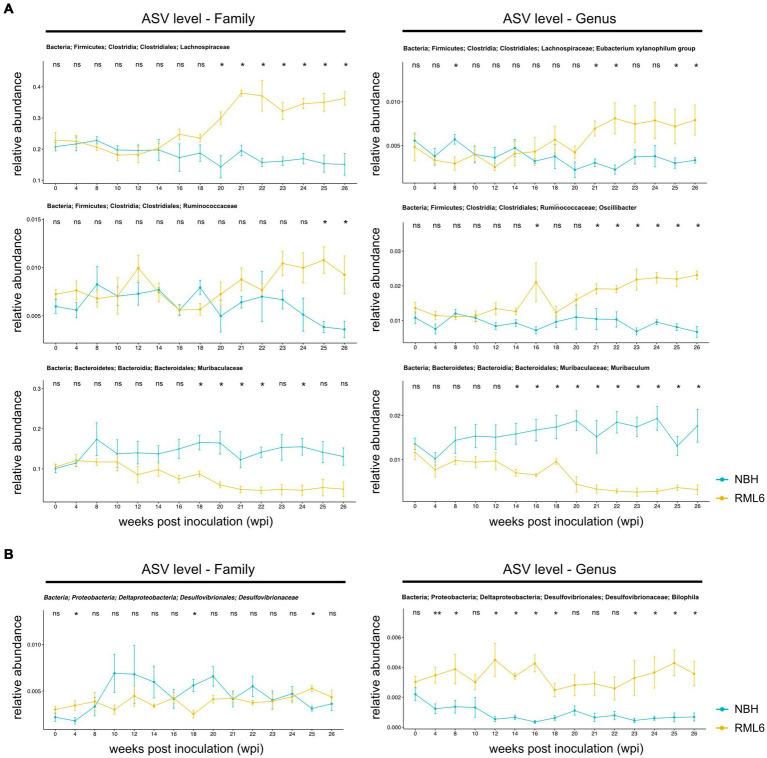
Relative abundance of significant gut microbes in prion disease. **(A)** Relative abundances of representative family (left panels) and associated genera (right panels) members. Within family, there are significant differences in clostridiales and bacteroidales between the control group (NBH) and prion (RML6)-infected animals. On the genera level, there are significant differences in abundance of Lachnospiraceae, Ruminococcaceae, and Muribaculaceae between control (NBH) and prion (RML6)-injected animals. Two-tailed Student’s *t*-test was performed. **(B)** Relative abundances of Desulfovibrionaceae family (left panel) and Bilophila genera (right panel). Bilophila show relative pre-symptomatic expansion starting 4 weeks post-prion inoculation as compared to NBH-inoculated mice. Pairwise comparison of each group at each time point and Holm–Bonferroni corrected for multiple comparisons. *p < 0.05; **p < 0.01; ns: not significant.

The bacterial taxonomy based on genome phylogeny represents a hierarchical structure consisting of seven levels (Parks et al., 2018). Accordingly, we analyzed the data to investigate the abundance of different microbiota in depth. First, we plotted all the detected families over time and condition in a heat map (Figure 2A). Second, we extended the analyses to all detected genera throughout the experimental groups (Figure 2B), allowing to potentially identify a prion disease-specific microbiome signature that could be exploited to develop early preclinical disease biomarkers. Overall, we detected *n* = 45 family and *n* = 106 corresponding genera members among the analyzed samples. Neurodegenerative diseases result in a cognitive decline, are often associated with movement disorders, decreased activity, and altered metabolic serum profiles eventually associated with microbiome structural changes (Erkkinen et al., 2018; Wang et al., 2021). Apart from one report describing that mice and humans carrying the AD high-risk Apolipoprotein E4 allele show an enrichment in Ruminococcaceae (Tran et al., 2019), little is known about the role of Lachnospiraceae and Ruminococcaceae in neurodegenerative diseases. When focusing on Lachnospiraceae and Ruminococcaceae, we detected a significant increase in the abundance of these taxa at the time point of symptom onset at week 20 in prion-diseased mice as compared to control mice (Figure 3A, upper and middle panels). These differences persisted, even with a slight upward trend, until the terminal stage of the disease. From this analysis, we conclude that our data are in line with the findings reported by others and that neurodegenerative proteinopathies relate to changes in microbiome structure throughout the course of prion disease as compared to control animals.

#### Diminished Muribaculum signature after prion disease symptom onset

Muribaculum has been described to play a protective role in the development of cognitive impairment in APP/PS1 transgenic mice, a murine model of AD. For instance, mice that had high microbial diversity food showed an improvement in spatial learning, objective recognition, memory as well as hippocampal Aβ plaque pathology as compared to APP/PS1 transgenic animals that were fed with aseptic food over the same period. Fecal 16S ribosomal RNA sequencing and analysis of these transgenic experimental mice revealed that cognitive function correlated positively with the presence of Muribaculum (Liu et al., 2020). Another group studied hippocampal gene expression in association with changes in microbial species in rats that were fed with a high-fat and high-sugar diet as such a diet leads to cognitive impairment over time (Leigh et al., 2020). A multiple regression analysis identified Muribaculum as a significant predictor of performance in the novel place recognition task. Therefore, we assessed microbiota and their relative abundance that have been reported to be positively and negatively associated with cognitive impairment, with a special focus on microbiome strains such as Muribaculum. At baseline, there was no difference between the experimental groups. After prion inoculations, the relative abundance of Muribaculum decreased over time. At week 14, upon prion infection (the time point when pre-symptomatic hippocampal gene expression changes occur), Muribaculum abundance differed significantly between control and prion-diseased mice. At week 18–20 post-prion inoculation, when disease symptoms manifest, the abundance of Muribaculum dropped markedly. Moreover, the abundance of Muribaculum slightly increased over time in NBH-injected control animals (Figure 3A, lower panel). These findings suggest that (intracerebral) prion pathology is associated with microbiome alterations and compositional shifts that favor and/or reflect cognitive impairment of prion disease in an anterograde manner.

#### Early preclinical intestinal Bilophila expansion in prion disease

As previously described, first disease symptoms typically occur at approximately week 20 post-prion inoculation (Sorce et al., 2020; Caredio et al., 2023). As it has been shown that intracerebral transcriptional and muscle metabolism changes already occur before the onset of disease symptoms (Sorce et al., 2020; Caredio et al., 2023), we examined whether there are microbiota that are either depleted or enriched in prion disease prior to and after the onset of disease symptoms. Relative to NBH mice, RML6-injected prion mice had a significant enrichment in relative abundance for Lachnospiraceae and Ruminococcceae. Lachnospiraceae were enriched after 14 wpi in the RML6-inoculated group, whereas Ruminococcaceae were enriched in the terminal stage of disease. On the other hand, Muribaculaceae were depleted in RML6 as compared to NBH control mice (Figure 3A, lower panels). By looking at the genera level, we consecutively identified several microbiotas that were either significantly enriched or depleted in RML6-infected mice compared to NBH-injected mice. After the onset of first clinical symptoms, Xylanophilum and Oscillibacter were significantly enriched in RML6 mice compared to NBH mice, whereas the opposite was true for Muribaculum (Figure 3A, upper and middle panels). Already at 4 wpi, and thus 16 weeks prior to any disease symptoms, Bilophila were significantly enriched in prion-infected animals (Figure 3B). Throughout the prion disease course, Bilophila were largely absent and not detectable in control mice, suggesting an association of this genera with neurodegeneration and prion disease pathogenesis. From this, we conclude that certain bacteria are enriched prior to disease symptoms, whereas others are enriched toward the terminal stage of disease. Our findings suggest that microbiome alterations occur even earlier in the disease pathogenesis than the previously described transcriptional intrahippocampal changes in prion-diseased animals (Sorce et al., 2020).

#### Functional metabolic pathway prediction advocates significant roles for lipid metabolism and short-chain fatty acids in prion disease

**Figure 4:**
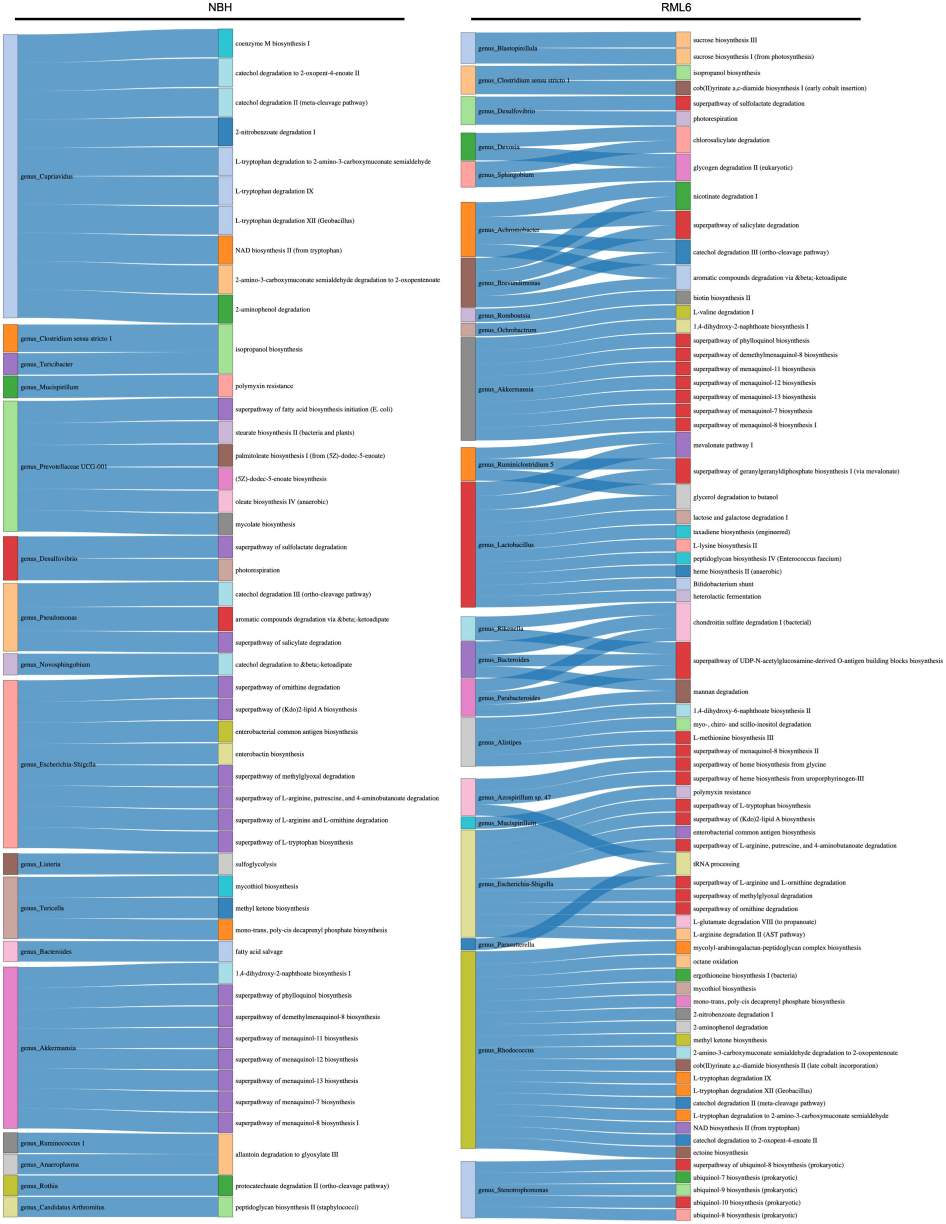
Functional metabolic prediction analysis in prion disease. Left column of both experimental conditions (NBH and RML6) represents the genus, and right column represents the metabolic pathway. Link between the left and right columns represents correlated terms.

**Figure 5:**
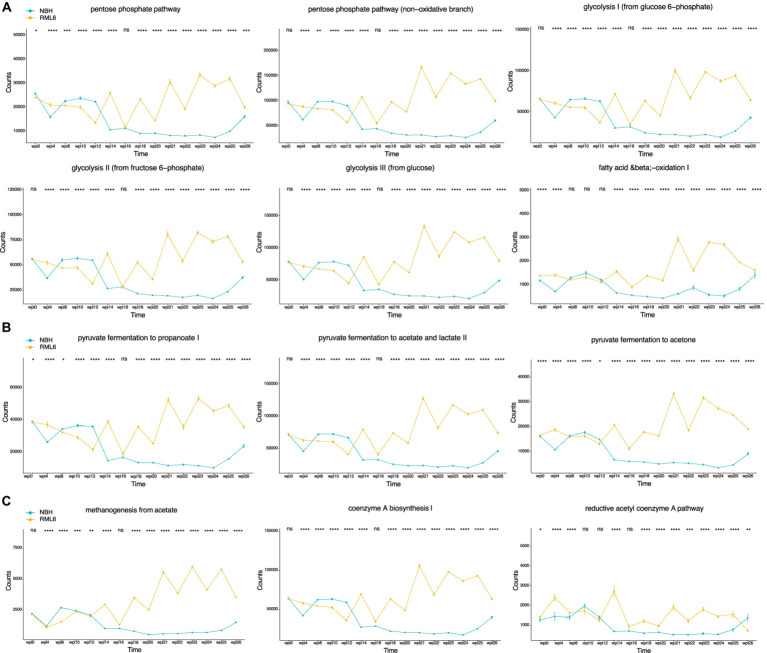
Temporal deconvolution of associated metabolic pathways based on relative microbiota abundance. **(A)** Pentose phosphate pathway, glycolysis, and fatty acid beta oxidation. **(B)** SCFAs, namely, acetate, propionate, and butyrate, are metabolized mainly from pyruvate, L-lysine, and acetyl-CoA fermentation. **(C)** Enrichment in methanogenesis (from acetate), coenzyme A biosynthesis, and acetyl coenzyme A pathway underlines the importance of SCFAs in prion disease. *p < 0.05; **p < 0.01; ***p < 0.001; ****p < 0.0001.

Gut bacteria can affect the lipid metabolism of the host (Jian et al., 2022; Brown et al., 2023). Short-chain fatty acids (SCFAs) and their main members, acetate, propionate, and butyrate, are synthesized via the Embden–Meyerhof–Parnas (glycolysis, for six-carbon sugars) and the pentose phosphate pathway (oxidative and non-oxidative branches for five-carbon sugars). They convert monosaccharides into phosphoenolpyruvate (PEP), followed by fermentation into these organic acids (Miller and Wolin, 1996; den Besten et al., 2013; Silva et al., 2020). SCFAs serve as energy sources, trophic intestinal factors, and regulators of regulatory T cells and exert crucial physiological effects on various organs, including the brain (Pascale et al., 2018; Dalile et al., 2019). Butyrate-producing bacteria include members of the families Ruminococcaceae and Lachnospiraceae (Louis and Flint, 2009; Louis et al., 2010; Louis and Flint, 2017; Parada Venegas et al., 2019). Both families were significantly more abundant in prion-infected animals (Figure 3). Moreover, it has been reported that butyrate promotes the fitness of bacteroidales, the most abundant human microbiome genus (Park et al., 2022). Therefore, we performed a functional metabolic prediction analysis based on relative bacterial abundances and 16S sequences to delineate pathways potentially relevant for prion disease pathogenesis. We first checked the main metabolic expressions being associated with genera and detected the absence of “fatty acid salvage” among bacteroides in RML6 in comparison with control mice, pointing to the importance of fatty acids in prion disease pathology (Figure 4). We then investigated the main pathways that yield SCFA precursors, namely pentose phosphate pathway, glycolysis, and fatty acid beta oxidation, as described above. All pathways were predicted to be enhanced in prion-diseased mice beyond 14 weeks of infection (Figure 5A). This prompted us to explicitly query for SCFA pathway expressions such as PEP and pyruvate (both derive from the above-mentioned pathways), which undergo fermentation into SCFAs and their precursors. Intriguingly, starting from week 14 upon prion infection, butanoate (butyrate), propanoate (propionate), acetate, lactate, and acetone fermentation (from pyruvate, L-lysine, and acetyl-CoA) were predicted to be upregulated in prion disease (Figure 5B). Moreover, methanogenesis (from acetate), coenzyme A biosynthesis, and acetyl coenzyme A pathways associated with SCFAs are predicted to be enriched in prion disease (Figure 5C). From these data, we learn that, in accordance with the associated microbiome landscape, the lipid metabolism and SCFA metabolic pathways are seemingly enhanced during prion disease course.

## Discussion

Even after decades of research, a cure for neurodegenerative disorders is still missing (Prusiner, 1998; Checkoway et al., 2011; Erkkinen et al., 2018). Here, we aimed to pursue an alternative approach and assessed the microbiome in prion disease. A better understanding of the role of the intestinal microbiome in the pathogenesis of CNS diseases may allow the identification of biomarkers, novel drug targets, and important pathways. In this context, targeting the microbiome appears promising as fecal microbiota transplantation (FMT) has successfully been applied to restore microbiome dysbiosis and alleviate Alzheimer’s disease-like pathogenesis *in vivo* (Sun et al., 2019).

To enhance the understanding of the interorgan crosstalk along the brain–gut–microbiome axis and its role in extra-intestinal CNS diseases such as prion disease, we investigated and characterized the temporal microbiome signature *in vivo*. It is now well recognized that a microbiome–brain crosstalk is essential in health and disease (Cryan et al., 2019; Nagpal and Cryan, 2021). The microbiome and its associated metabolites are not only crucial for peripheral immune homeostasis but are also paramount for immune cells, neuronal circuits, and brain function (Strandwitz, 2018; Lai et al., 2021; Yu and Hsiao, 2021). Moreover, it is recognized that microbiome alterations are associated with CNS and extra-axial pathologies such as autoimmune, neuroinflammatory, neuropsychiatric, and neurodegenerative diseases (Scher et al., 2013; Tremlett et al., 2016; Lach et al., 2018; Nagpal and Cryan, 2021). To determine the effects of altered cerebral homeostasis on microbiome composition, we analyzed fecal specimen on a weekly basis, assessed body weight changes, and performed a clinical severity scoring system throughout the disease course.

The brainstem harbors several critical neuronal structures, including motor and autonomic centers, the locus coeruleus, and the nucleus of the solitary tract, from where vagus nerve fibers originate. These structures were shown to be delicate areas in prion disease pathology (Mirabile et al., 2015). We, therefore, first confirmed the presence of prion aggregates throughout the brain, including the cortex and brainstem, upon euthanasia. Presumably, microglia serve as relays in the brain–gut axis as they carry out broad responsibilities in balancing and restoring host homeostasis, influencing synaptic remodeling, and communicating with afferent and efferent peripheral neurons (Abdel-Haq et al., 2019; Defaye et al., 2022). It is therefore conceivable that prion aggregate-induced microglia activation (Aguzzi and Zhu, 2017; Aguzzi, 2022) mediates an altered bidirectional neuronal (i.e., vagus nerve-mediated) crosstalk between anatomical regions of the brain(stem) and the intestine (Savchenko et al., 2000; Badoer, 2010; Hickman et al., 2018).

To determine whether microbiome perturbations occur in intracerebrally inoculated prion-diseased animals, we assessed microbiota diversity using alpha and beta diversity based on their relative abundance and observed diversity changes. Especially beta diversity was significantly changed in relation to disease state. It is likely that the large standard deviations are the main contributor to the overall non-significant change in alpha diversity. We then checked for differences in relative abundance among various taxonomy levels and found significant differences in preclinical, symptomatic, and terminal disease stages. In prion-diseased animals, we detected a significant enrichment in Lachnospiraceae, Desulfovibrionaceae, and Ruminococcaceae and a depletion of Muricaculaceae. Intriguingly, data from patients suffering from MS, an autoimmune neuroinflammation disorder, show comparable alterations among these taxa members (Tremlett et al., 2016). As described above, data suggest that Muribaculum correlates positively with cognitive function (Leigh et al., 2020; Liu et al., 2020). The identification of Muribaculum reduction in prion-infected animals at week 14 of disease course might indeed reflect a temporal association between hippocampal gene expression changes and microbiota colonization. The discovery of Bilophila enrichment 4 weeks after prion inoculation and thus long before the manifestation of any clinical symptoms was unexpected. It has been described that preclinical hippocampal transcriptional and muscle metabolism changes occur 8 weeks post-prion infection and therefore well before the onset of any clinical symptoms, which normally occur 20 weeks after prion inoculation (Sorce et al., 2020; Caredio et al., 2023). Our finding that Bilophila abundance changes 4 weeks post-prion inoculation suggests that peripheral and metabolic disease-associated alterations, including intestinal microbiome changes, occur even earlier than previously appreciated. To this end, we investigated the metabolic profile, calculated based on the apparent murine microbiome constituents with a focus on fatty acid metabolism, and identified enhanced phosphate pentose pathway, glycolysis, and beta oxidation metabolic processes in prion disease. In general, upregulation of these pathways results in elevated levels of PEP and pyruvate molecules that can undergo fermentation into SCFAs (Fernie et al., 2004; den Besten et al., 2013). It might therefore be conceivable that upregulated bacterial metabolic pathways mirror additional energy needs in the setting of neuroinflammation or that upregulation of SCFA synthesis reflects an effort to maintain immune cell capacity to dampen pathogenic and inflammatory processes, with the aim to restore central immune homeostasis by influencing microglia activity via meningeal immune cell interaction (Rothhammer et al., 2016; Martin-Pena and Tansey, 2023; Seo et al., 2023). Butyrate-producing bacteria include Ruminococcaceae and Lachnospiraceae members (Louis and Flint, 2009; Louis et al., 2010; Louis and Flint, 2017; Parada Venegas et al., 2019), whereas it has been shown that the presence of Bilophila (*wadsworthia*) influences SCFA synthesis toward suppressed fecal SCFA concentrations (Natividad et al., 2018). Moreover, Bilophila monocolonization increases susceptibility to cognitive impairment by disrupting hippocampal synaptic plasticity and neurogenesis in mice (Olson et al., 2021). Therefore, our data suggest that the relative increase in Lachnospiraceae and Ruminococcaceae, but not Bilophila, might indeed be responsible for enhanced SCFA production in prion disease. As Lachnospiraceae have also been linked to other neurodegenerative diseases, including PD, it is possible that this family contributes to a variety of protein misfolding diseases in a more unspecific manner (Vascellari et al., 2020). Although it has recently been reported that prion disease pathogenesis was not worsened in germ-free mice (Bradford et al., 2017), we report significant dynamic microbiome compositional and metabolic perturbations in prion-diseased animals, supporting the relevance of a brain–gut–microbiome mechanism of unclear significance. The fact that antimicrobial agents such as doxycycline have been tested successfully in preclinical and clinical settings in prion disease therapy and prevention further supports the relevance and involvement of the microbiome in prion pathology (Stewart et al., 2008; Forloni et al., 2019). Mechanistically, it may be that prion-induced and microglia-derived molecules as well as altered neurotransmitter signaling result in a less stringent immune-controlled gut microbial environment, possibly mediated by an aberrant vagus nerve or circulatory signaling, in line with the fact that central neuronal loss and gliosis disturb the brain–gut axis (Abdel-Haq et al., 2019; Cryan et al., 2019). Another route of action might be that brainstem-mediated imbalances in vagus nerve function influence intestinal glial cells since this cell population has been shown to govern intestinal motility at the level of the myenteric plexus (Rao et al., 2017). Finally, it is also possible that altered movement and eating behaviors, both occurring at different stages of neurodegenerative diseases (Guenther et al., 2001; Fostinelli et al., 2020), result in metabolic changes that favor perturbations in the microbiome.

## Conclusion

We observed perturbations in the gut microbiome of mice throughout prion disease course in a temporal manner. These alterations were characterized by taxonomic depletions and enrichments in a limited number of microbial taxa rather than colossal compositional shifts as changes were only significantly evident at beta diversity level. This study suggests that neuroinflammatory processes are associated with an impaired brain–gut–microbiome axis and that microbiome and metabolic changes occur prior to clinical symptoms. Finally, we speculate that our findings might have diagnostic and therapeutic implications as microbiota and their metabolites could serve as early disease and therapy monitoring biomarkers or as targets for disease-modifying therapies that aim to ameliorate inflammation and disease progression in humans. It is conceivable that targeting the microbiome and related metabolites not only has implications on immunotherapy responses in malignancies but also in other conditions along the gut–brain axis.

## Limitations and potential future directions

Despite the description of a dynamic temporal microbiome signature and underlying altered metabolic pathways in prion disease, additional work is required to resolve precise molecular mechanisms involved. Although the findings of this study hold promise for treating and anticipating development of prion disease, discoveries need to be corroborated and studied in human patients suffering from prion disease at present, and optimally also in individuals with a positive prion disease family history to assess translational perspectives. In addition to our intracerebral inoculation prion model that involves Brain stem pathology, future studies that may focus on the myenteric plexus–microbiome crosstalk may also consider harnessing the intraperitoneal route of prion infection. Whether the findings reported here indicate a specific microbiome signature of prion disease or of broader neuroinflammatory diseases and processes remains to be determined.

## Materials and methods

### Mice

Animal care and experimental protocols were in accordance with the Swiss Animal Protection Law. Mice were bred in a high hygienic-grade facility of the University Hospital of Zurich (BZL), housed in groups of 2–5, and monitored for pathogens such as bacteria, parasites, and viruses as described in the Federation of European Laboratory Animal Associations (FELASA). Individual housing was avoided in the best possible way. The light/dark cycle of mice consisted of 12/12 h with artificial light from 7 a.m. to 7 p.m. at 21 ± 1°C. Animals were fed with Kliba-Nafag (3242.PX.S12, vitamin-fortified, irradiated). C57BL/6/J male wild-type mice were obtained from Charles River (Germany). We performed the experiments with the highest possible age congruence among the experimental (RML and NBH) groups. Mice were randomly assigned to the experiments. Mice from these experimental cohorts were also used for a ribosomal profiling study in prion disease (Scheckel et al., 2020). Therefore, the decrease in animal numbers over time was intentional.

### Prion inoculation, disease symptoms, and severity assessment

Six- to 10-week-old male mice were intracerebrally (right hemisphere between eye and ear) injected with 30 μL of RML6 prions (passage 6 of Rocky Mountain Laboratory strain mouse-adapted scrapie prions) containing 8.02 log LD_50_ of infectious units per ml. Control mice were inoculated with 30 μL of non-infectious brain homogenate (NBH) at the same dilution. Upon inoculation, mice were monitored three times per week up to the first clinical symptoms and scored with a clinical severity score. After the clinical onset, mice were monitored daily. Mice were sacrificed at respective time points corresponding to an age of 13–26 weeks. The scoring system consisted of two categories: (A) Grooming and (B) Activity/Ataxia/Posture from which each category (A and B) has a possible score of 0–5 points. The clinical severity score results from the sum of categories A and B. Category A—0: shiny, well-kept hair coat; 1: signs of reduced grooming; 2: dull coat; 3: ungroomed coat; 4: piloerection; 5: discharge from eyes. Category B—0: normal moving and no sign of kyphosis; 1: occasionally limp while walking and reduced nest-building activity; 2: limping and mild kyphosis but able to straighten its spine; 3: rarely tumbles, able to position itself correctly, shows mild but persistent kyphosis and inability to straighten its spine completely; 4: tumbling, able to position itself correctly, persistent kyphosis and leg stiffness while walking; 5: severe activity reduction. Mice are directly euthanized upon clinical symptoms of the terminal disease stage, such as persistent kyphosis, severe ataxia, hind-limb clasping, and piloerection.

### Fecal specimen collection

Once a week, stool specimens were harvested from all experimental and control cages at the same time point. Upon collection, specimens were snap-frozen in liquid nitrogen and stored at −80°C until sequencing preparations. Mice were euthanized using carbon dioxide and decapitation upon anesthesia. For histology and immunohistochemistry, brains were immediately dissected and fixed in 4% formalin at 4°C overnight, followed by a prion decontamination step (1-h formic acid treatment at RT followed by a 2-h formalin post-fixation step) before paraffin embedding. Fecal specimens were sent for sequencing according to the guidelines of Microsynth (Switzerland).

### 16S ribosomal RNA sequencing and amplicon sequence variant analysis

Illumina Nextera barcoded, two-step PCR libraries (16S V4) were used to build up the library, and Illumina MiSeq, v2, 2x250bp was used for sequencing. We used the QIIME2 pipeline for the analysis of 16S rRNA (Bolyen et al., 2019). After checking data quality, denoising with DADA2 was performed to merge the paired reads, generating amplicon sequence variants (ASVs) (Callahan et al., 2016). Alpha rarefaction module was used to ensure that we had sufficient depth to capture most features. We then calculated both alpha and beta diversity using the core-metrics-phylogenetic module. For alpha analysis, we used the faith phylogenetic matrix to compute the richness and incorporate features of phylogenetic relations. In beta diversity, we used the weighted UniFrac distance matrix to quantify the dissimilarity between communities (Diallo et al., 2007). Principal coordinate analysis (PCoA) was used for better visualization of beta diversity. Taxonomy was assigned to ASVs using the q2-feature-classifier classify-sklearn naïve Bayes taxonomy classifier against the pre-trained Naïve Bayes silva-132-99-nb-classifier trained against Silva (release 132) full-length sequences (Bokulich et al., 2018). Taxa barplot module was used for the visualization of the different taxonomy composition.

### Functional metabolic pathway prediction

To predict metagenome functions and pathway abundance based on 16S amplicon sequences, we used the PICRUSt2 algorithm (Douglas et al., 2020). In short, genus and pathway abundance were prepared upon sample aggregation (NBH and RML6) across different time points (wpi 0–26). Correlation was run between genus and pathways within each condition, followed by filtering the significant correlation with a value less than 0.001 and a correlation value more or less than 0.7. Since this might lead to multiple correlations (one genus could correlate with more than one pathway), Sankey plots were plotted for each experimental condition separately for data visualization and interpretation. Assessment of significant metabolic processes between experimental conditions (NBH vs. RML6) and their temporal dimension was performed.

### Histology and immunohistochemistry

Formalin-fixed, formic acid-treated, and paraffin-embedded brain sections (2–4 μm) were deparaffinized with 3 cycles of xylene treatment followed by re-hydration with 100% EtOH, 96% EtOH, 70% EtOH, and water (each treatment 5 min), respectively. Hematoxylin and eosin (HE) stain and immunohistochemistry (IHC) for SAF84 were performed according to standard procedures at our institute. In short, deparaffinized sections were treated with formaldehyde for 30 min followed by 98% formic acid treatment for 6 min and then washed with distilled water for 30 min. After incubation with protease 1 (Ventana) for 16 min, sections were incubated with polyclonal anti-PrP antibody (SAF84, SPI Bio) at a dilution of 1:200. Sections were counterstained with hematoxylin. An Olympus BX61 system was used to acquire images.

### Statistical analysis

A two-way analysis of variance (ANOVA) was conducted to compare the relative abundance of taxa between two groups (NBH and RML6) at multiple time points. The pairwise comparison of each group at each time point was also performed and corrected for multiple comparisons using the Holm–Bonferroni method. A *p*-value of less than 0.05 was considered significant. Additionally, a pairwise PERMANOVA of beta diversity was performed to assess differences in overall community composition between the two groups at each time point. The PERMANOVA was performed using the “adonis” function in the “vegan” package in R. All statistical analyses were performed using the R statistical software. The two-way ANOVA was used to determine the significance of the effect of both the treatment (NBH vs. RML6) and time point on the relative abundance of taxa, while the pairwise comparisons were used to identify which specific time points showed significant differences between the two groups. The PERMANOVA was used to assess the significance of differences in overall community composition between the two groups at each time point. The Holm-Bonferroni correction was applied to control the inflation of type I errors due to multiple comparisons.

## Data availability statement

The original contributions presented in the study are publicly available. This data can be found here: BioProject, accession number: PRJNA1114866.

## Ethics statement

The animal study was approved by animal care and experimental protocols were in accordance with the Swiss Animal Protection Law and approved by the Veterinary office of the Canton of Zurich, Switzerland (permits ZH040-15, ZH243-18). The study was conducted in accordance with the local legislation and institutional requirements.

## Author contributions

ML: Conceptualization, Data curation, Investigation, Methodology, Project administration, Resources, Validation, Visualization, Writing – original draft, Writing – review & editing. YM: Data curation, Formal analysis, Methodology, Software, Validation, Visualization, Writing – review & editing. ME: Writing – review & editing. SM: Writing – review & editing. PS: Project administration, Resources, Writing – review & editing. AA: Funding acquisition, Resources, Writing – review & editing. MS: Conceptualization, Funding acquisition, Project administration, Resources, Supervision, Writing – review & editing.
