# Supplementary material for: Longitudinal microbiome investigation throughout prion disease course reveals pre- and symptomatic compositional perturbations linked to short-chain fatty acid metabolism and cognitive impairment in mice
Source: Front Microbiol. 2024 Jun 11;15:1412765. doi: 10.3389/fmicb.2024.1412765 (PMC11196846; doi:10.3389/fmicb.2024.1412765)
Supplement: Supplementary file 1 [file Table_1.pdf]

Supplementary material, Losa et al., fmicb, 2024

Table S1. *Age distribution at inoculation among male experimental mice.*

Experimental groups	**NBH**	**RML6**	**Sign.**
Age in weeks (mean+/- SEM)	27.1 +/- 1.75	26.58 +/- 1.67	ns
