# Supplementary material for: Longitudinal microbiome investigation throughout prion disease course reveals pre- and symptomatic compositional perturbations linked to short-chain fatty acid metabolism and cognitive impairment in mice
Source: Front Microbiol. 2024 Jun 11;15:1412765. doi: 10.3389/fmicb.2024.1412765 (PMC11196846; doi:10.3389/fmicb.2024.1412765)
Supplement: Supplementary file 2 [file Table_2.pdf]

Supplementary material, Losa et al., fmicb, 2024

Table S2. *Animal numbers and cages of experimental groups.*

Timepoint (wpi)	**0**	**4**	**8**	**10**	**12**	**14**	**16**	**18**	**20**	**21**	**22**	**23**	**24**	**25**	**26**
Mice (=n) RML6	21	21	18	18	15	15	15	13	13	13	12	12	12	12	9
Cages (=n) RML6	5	5	5	5	4	4	4	4	4	4	4	4	4	4	4
Mice (=n) NBH	21	21	18	18	15	15	15	13	13	13	12	13	13	13	13
Cages (=n) NBH	5	5	5	5	4	4	4	4	4	4	4	4	4	4	4
