# Supplementary material for: Longitudinal microbiome investigation throughout prion disease course reveals pre- and symptomatic compositional perturbations linked to short-chain fatty acid metabolism and cognitive impairment in mice
Source: Front Microbiol. 2024 Jun 11;15:1412765. doi: 10.3389/fmicb.2024.1412765 (PMC11196846; doi:10.3389/fmicb.2024.1412765)
Supplement: Supplementary file 3 [file Table_3.pdf]

Supplementary material, Losa et al., fmicb, 2024

Table S3. *Statistical values of the PCoA resulting from all analyzed experimental timepoints.*

Timepoint (wpi)	Permanova *P*-value
**0**	0.83
**4**	0.306
**8**	0.298
**10**	0.028
**12**	0.084
**14**	0.032
**16**	0.215
**18**	0.033
**20**	0.022
**21**	0.029
**22**	0.021
**23**	0.024
**24**	0.029
**25**	0.029
**26**	0.031
