# Supplementary material for: Longitudinal microbiome investigation throughout prion disease course reveals pre- and symptomatic compositional perturbations linked to short-chain fatty acid metabolism and cognitive impairment in mice
Source: Front Microbiol. 2024 Jun 11;15:1412765. doi: 10.3389/fmicb.2024.1412765 (PMC11196846; doi:10.3389/fmicb.2024.1412765)
Supplement: Supplementary file 4 [file Image_1.pdf]

Supplementary material, Losa et al., fmicb, 2024

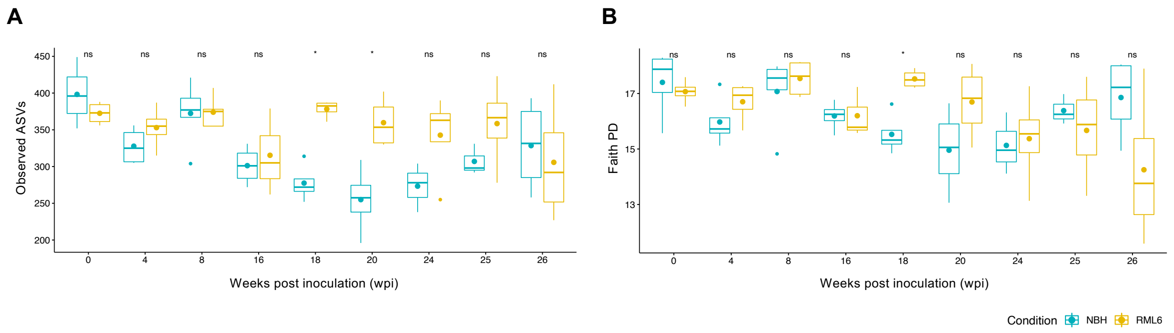

Figure S1. Alpha diversity between NBH and RML6 across different time points after prion infection. **(A)** The number of observed amplicon sequence variants (ASVs) and **(B)** Faith phylogeny degree (PD) alpha matrices over time. Diversity analysis reveals microbiome behavioral changes between experimental groups from 18 wpi. **P*<0.05; ns: not significant. Pairwise comparison of each group at each time points and Holm-Bonferroni corrected for multiple comparisons.
